# Ruminal Fermentation, Growth Rate and Methane Production in Sheep Fed Diets Including White Clover, Soybean Meal or *Porphyra* sp.

**DOI:** 10.3390/ani10010079

**Published:** 2020-01-02

**Authors:** Vibeke Lind, Martin R. Weisbjerg, Grete M. Jørgensen, Júlia E. Fernandez-Yepes, Lesly Arbesú, Eduarda Molina-Alcaide

**Affiliations:** 1 Norwegian Institute of Bioeconomy Research (NIBIO), PB 115, N-1431 Aas, Norway; grete.jorgensen@nibio.no; 2 Department of Animal Science, Aarhus University, AU Foulum, Blichers Allé 20, 8830 Tjele, Denmark; martin.weisbjerg@anis.au.dk; 3 Estación Experimental del Zaidin (Consejo Superior de Investigaciones Científicas), Profesor Albareda, 1, 18008 Granada, Spain; julia.fernandez@eez.csic.es (J.E.F.-Y.); lesly.arbesu@eez.csic.es (L.A.); molina@eez.csic.es (E.M.-A.)

**Keywords:** Macroalgae, lamb, ewe, in vitro, in vivo, performance, seaweed

## Abstract

**Simple Summary:**

In ruminant feeding, the use of diets containing seaweeds could be a valuable alternative to conventional diets. The objective of this work was to investigate the ruminal fermentation, growth rate and methane production in sheep fed a diet including *Porphyra* sp. compared with diets including clover silage or soybean meal. Including *Porphyra* sp. had little impact on ruminal fermentation and methane production both in vitro and in vivo. Lambs fed *Porphyra* sp. had a similar growth rate to those fed a diet including soybean meal, confirming previous in vitro and in situ observations on the high-quality protein of *Porphyra* sp. in ruminant feed.

**Abstract:**

The aim of the present work was to investigate the potential of *Porphyra* sp. as an alternative source of protein to soybean meal in diets for sheep. Our experimental treatments included a control diet (CON) based on grass silage and crushed oats and three diets containing protein supplements, clover silage (CLO), soybean meal (SOY) or *Porphyra* sp. (POR) to increase dietary crude protein concentrations. We studied its effects on rumen fermentation, growth rate and methane emissions. Ruminal fermentation characteristics, kinetics of gas production and methane production were studied in vitro by using batch cultures inoculated with rumen inoculum from sheep. There were no differences among diets in total volatile fatty acids (VFA) production or in the VFA profile in vitro. Across treatments, we measured no differences in methane production either in vitro or in vivo, and we saw no noticeable antimethanogenic effect of *Porphyra* sp. The present in vivo trial with lambs showed no differences in average daily weight gain when fed diets including *Porphyra* sp. or soybean meal diets (250 and 254 g/d, respectively). We conclude that *Porphyra* sp. has a protein value similar to high-quality protein sources like soybean meal.

## 1. Introduction

The increased demand for food to provide for a growing global population, estimated to reach more than 9 billion by 2050 [1], is a challenge for animal production systems. Prohibition of the use of animal and fish protein subsequent to the BSE crisis in the 1990s, along with structural changes in cereal production at the expense of grain–legumes [2], produced a gap in the supply of protein to ruminants. Additional protein was needed and imported soybean meal seemed to be the most suitable. Norwegian cereal and seed production do not meet the feed demands for increased animal production. Thus, more than 90% of the protein and 40% of all the ingredients in Norwegian commercial concentrates are imported [3]. In addition, since 1996, the import and use of GMO-ingredients are prohibited [4]. Therefore, in Norway, a focus on locally produced concentrates covering the demand for protein sources is essential.

Traditionally, seaweed has been used in Norwegian coastal communities as a feed supplement, especially when there was shortage of forage [5]. Due to geography, environment and the characteristics of aquaculture production, conditions along the Norwegian coast are well suited to produce seaweed. 

The protein concentration is high in *Rhodophytal* (red) and *Chlorophyta* (green) seaweed species (10–47% of dry matter (DM)) compared to that in *Phaeophyta* (brown) seaweed species (3–15% of DM) [6,7,8,9]. For the red seaweed *Porphyra* sp., the protein concentration may be as high as 47% of DM in the spring, which could make it relevant as a substitute for soybean meal [7]. In general, the amino acid composition of *Porphyra* sp., is similar to proteins from soybean and eggs [9,10,11]. Palatability when feeding seaweeds to ruminants has been mentioned by, e.g., Roque et al. [12] and Özkan-Gülzari et al. [13] and dealt with by adding water or molasses.

It has long been accepted that the feeding value of protein rich legumes is often greater than that of grasses fed to ruminants [14]. This is because of their more rapid particle breakdown, faster rumen fermentation, lower mean rumen retention time and, consequently, greater voluntary feed intake [15]. White clover (*Trifolium repens*) contains from 17% to 33% protein, depending on its stage of maturity, and is an important source of protein.

Enteric emissions of methane (CH_4_) by ruminants, arise primarily from the fermentation of feed in the rumen. Methane is an important greenhouse gas and represents an energy loss from the animal that results in reduced productivity. Because methane emissions are influenced by type of feed, ruminant nutritionists have focused studies on dietary strategies that can reduce CH_4_ emissions [16,17,18].

In vitro and in situ techniques are used in animal nutrition research as they are less expensive, time-consuming and laborious compared to in vivo experiments. There are some in vitro and in situ studies investigating the nutritive value of seaweeds [7,8,9,19] but, to our knowledge, very few in vivo experiments have been performed using seaweed as a protein source for ruminants [12,20,21]. Also, we have no knowledge of in vivo experiments including *Porphyra* sp. to investigate enteric CH_4_ production or growth rate in ruminants. Therefore, *Porphyra* sp., the most promising seaweed of eight Norwegian species tested in vitro and in situ by Tayyab et al. [7], Molina-Alcaide et al. [8] and Gaillard et al. [9], was used in the present work. In this study, we compared the effect of dietary protein source on animals’ ruminal fermentation, growth rate and CH_4_ production both in vitro and in vivo. In particular, our aim was to assess the efficacy of *Porphyra* sp. as a protein source when compared with more traditional sources such as clover silage and soybean. We hypothesized that *Porphyra* sp. can substitute soybean meal or clover silage as a protein source in sheep diets without affecting ruminal fermentation, growth rate or enteric methane production.

## 2. Materials and Methods

### 2.1. Diet Ingredients

We used the following ingredients in our diets: first cut grass silage (*Bromus inermis, Poa pratensis, Festuca pratensis*), first cut white clover (*Trifolium repens* Litago) silage, crushed oats, soybean meal and the seaweed *Porphyra* sp. (CoDO International limited Yantai City, China), in addition to a standard mineral–vitamin mixture (PLUSS sau pellets, Felleskjøpet Agri, Gardermoen, Norway). The chemical composition is shown in Table 1. Both grass and white clover were wilted for one day after cutting before packing in round bales without any additives. The fermentation quality of the silages was good (pH 4.2 for both silages) and levels of lactic, acetic, propionic and butyric acid were within normal ranges according to Eurofins [22]. *Porphyra* sp. is not yet cultivated in Norway and therefore manual collection for in vivo trials would be too labor intensive and time consuming. Chemical analysis of the purchased *Porphyra* was compared to *Porphyra* sp. collected from the Norwegian coast and found to be similar (35.5 and 34.6 g/100 g crude protein, respectively). *Porphyra* sp. was purchased in the form of powder (dried and milled) and used without further processing. The product was stored in a clean and dry place until use.

### 2.2. Diets

A control diet (CON) based on grass silage and crushed oats was compared with three experimental diets with an additional protein source of either clover silage (CLO) or soybean meal (SOY) or *Porphyra* sp. (POR). All diets included the vitamin–mineral mixture. The ingredients and chemical compositions of the diets are shown in Table 2. The proportion of white clover in the CLO diet was 59% while the proportion of the protein sources soybean meal and *Porphyra* sp., in the SOY and POR diets, respectively, constituted about 10% of the diet.

The DM content of the four diets was around 88% and the crude protein content varied from 111 g/kg DM (CON) to 125, 137 and 126 g/kg DM for CLO, SOY and POR diets, respectively. Diets with protein enrichment (CLO, SOY and POR) were composed to be isoenergetic and isoproteinic.

### 2.3. In Vitro Trial

Three adult, dry, non-pregnant and rumen-fistulated Segureña sheep (Mean ± SD; 51.9 ± 2.75 kg body weight (BW)) were used as inoculum donors for the in vitro incubations. Animals were placed in individual boxes and fed alfalfa hay supplied to meet energy maintenance requirements [23]. Animals had free access to water, were cared for and handled by trained personnel in accordance with the Spanish guidelines for experimental animal protection (Royal Decree 53/2013 of February 1st on the protection of animals used for experimentation or other scientific purposes). All the experimental procedures were approved by the Animal Welfare Committee at the Estación Experimental del Zaidin (CSIC, Spain).

The batch culture procedure was used to study ruminal fermentation characteristics, CH_4_ emissions and the kinetics of gas production from control and experimental diets. The experimental procedure was based on the Theodorou et al. [24] protocol with some modifications. Rumen content from each of the three sheep was collected before the morning feeding, pooled and immediately taken to the laboratory into thermal flasks. Rumen contents were strained through four layers of cheesecloth and mixed with a buffer solution [25] in a 1:4 ratio (vol/vol) at 39 °C under continuous flushing with CO_2_. The time required from rumen content collection to inoculation of bottles was <30 min. Control and experimental diets were mixed in rations similar to the in vivo trial. Samples of the diets were ground (1 mm) before analyses for chemical composition and used as substrates in the in vitro runs. Three identical 72 h incubation runs were carried out over three consecutive weeks. Four bottles per diet and four bottles without added diet (blanks) were incubated in each run. Blanks were used to correct the gas production values for gas release from endogenous substrates. Samples (0.500 g) of diets were carefully weighed into 120 mL bottles, and 60 mL of the buffered rumen fluid was anaerobically added into each bottle. Bottles were sealed with butyl rubber stoppers and aluminum caps and incubated at 39 °C in a water bath. In two of the four bottles for each diet and two blanks, pressure and gas volume were measured at 2, 4, 6, 8, 12, 24, 48 and 72 h of incubation using a Wide Range Pressure Meter (Sper Scientific LTD, Scottsdale, AZ, USA) and a glass-calibrated syringe (Ruthe^®^, Normax, Marinha Grande, Portugal), respectively. In the remaining two bottles of each sample, the gas produced after 24 h of incubation was measured as described above and a gas sample (about 5 mL) was stored in an evacuated tube (Terumo Europe N.V., Leuven, Belgium) for the analysis of CH_4_. Bottles were then uncapped, the pH was measured immediately (Crison Basic 20 pH-meter, Crisson Instruments, Barcelona, Spain), the fermentation was stopped by placing the bottles in ice water, and the following samples were taken: 2 mL was added to 2 mL of deproteinising solution (20 g of metaphosphoric acid and 0.6 g of crotonic acid per litre) for volatile fatty acid (VFA) determination and 1 mL was mixed with 1 mL 0.5 M HCl for NH_3_-N analysis.

### 2.4. In Vivo Trials

The in vivo trials were conducted in accordance with the regulation for use of animals in experiments, adopted by the Norwegian Ministry of Agriculture and Food, and approved by the Ethics Commission on Animal Use by the Norwegian Food and Safety Authority, application number FOTS ID 8836. It complies with the EU Directive 2010/63/EU on the use of experimental animals, which was incorporated to the European Economic Area Agreement in May 2015.

#### 2.4.1. In Vivo Experiment 1

Forty-eight Norwegian White sheep ewes (average BW ± SEM of 52.6 ± 4.1 kg) were used in two replicates with 24 animals per replicate. For each replicate, the animals were distributed into six blocks according to initial weight, with the four lightest animals (block 1) randomly allocated to one of the four diets. Block 2 consisted of the next four lightest lambs and so forth until Block 6, which consisted of the four heaviest lambs. Within each block, animals were randomly allocated to one of the four diets studied. The ewes were stalled individually in an uninsulated barn at NIBIO Tjøtta in Nordland county, Norway (65°49′22 N 12°25′37 E) with free access to water. Animals were fed their daily allowances in two equal portions at 08:30 and 14:30 h. Grass silage was weighed daily and supplied ad libitum allowing a minimum of 10% leftovers. Leftover grass silage and white clover silage was measured daily, and daily intake calculated. Because the animals were skeptical regarding intake of the powdered *Porphyra* sp., it was mixed to a “porridge” consistency with the crushed oats, the mineral and vitamin mixture and 5 dl of cold water. Samples of grass and clover silages were collected weekly during the trial, and frozen at −18 °C for later analysis. Samples of the other ingredients (oats, soybean pellets and *Porphyra* sp.) were collected every second week and pooled for analysis.

Within each replicate, ewe’s adaptation to their corresponding diets before entering the respiration chambers were allowed over a period of 20 to 27 days, due to their staggered entry to chambers. The six open circuit respiration chambers at NIBIO Tjøtta have a 25 × 25 mm frame of coated steel covered with a steel mesh, that protects the exterior polycarbonate sheeting [26]. Each chamber measured 1.8 m wide, 1.8 m deep and 1.5 m high, making a total volume of approximately 5 m^3^. During the first replicate, enteric CH_4_ was sampled via plastic tubes into a seven port MGA 3500 multigas analyzer (ADC Gas Analysis Ltd., Hoddesdon, UK). Due to machine failure, the second replicate was sampled into a seven port Servopro Multiexact 4100 Analyzer (Servomex Group Inc., Woburn, MA, USA). Methane concentrations in ambient air and in exhaust gas leaving each chamber (in total seven ports) were measured on a rotational basis, taking one gas sample reading after 3 min per port, measuring ambient air and each chamber every 21 min. All diets were represented in all chambers equally. The animals were kept individually in the chambers for 72 h, during which they were fed and managed in a similar way as in the barn. Feed intake was measured daily. Water was available in buckets. Chambers were cleaned daily during morning feeding.

#### 2.4.2. In Vivo Experiment 2

Twenty-four Norwegian White lambs at an age of five months and average BW ± SEM of 32.9 ± 0.3 kg were used in the experiment. The animals were blocked according to initial weight, as described in in vivo Experiment 1. The lambs were stalled individually in the uninsulated barn at NIBIO Tjøtta with free access to water and fed as described in in vivo Experiment 1. Lambs were weighed regularly throughout the trial to calculate individual growth rate and adjust feed intake to their requirements. The lambs were adapted to their diet during a 20-day period followed by a recording period of six weeks. Animals’ weight gain was calculated based on start weight and weight after the six weeks recording period. Feed intake was measured four days every week during the six weeks of recording. After adaptation to the corresponding diet, two lambs within each diet were randomly picked for rumen fluid sampling, making up a total of eight samples. A short, rigid PVC tube of 12 mm (Tricoflex) was inserted in the esophagus of the animal before a 6 mm flexible PVC tube (Tricoflex) with about 12 holes of 3 mm diameter was inserted into the rumen to a depth of approximately 120 cm via the plastic tube. Rumen samples (ca. 50 mL) were obtained using an electric vacuum pump (Edwards RV5, serial no 119464845, West Sussex, UK). The rumen samples were examined visually to ensure they were not contaminated with saliva. The samples were strained through a layer of leno bandage (Mesoft, Mölnlycke health care, Göteborg, Sweden) and pH values were measured immediately using a pH-meter (VWR International pHenomenal, PC 5000 H, Serial no 11232089, Leuven, Belgium) following the methodology described by Ramos-Morales et al. [27]. Four milliliters of rumen filtrate were acidified with 4 mL of 0.5 M HCL for ammonia determinations and another 4 mL sample was deproteinized with 4 mL of a crotonic solution (250 mL HCL 0.5 M, 0.5 g crotonic acid and 5 g of metaphosphoric acid) for determination of acetate concentration. All samples were freeze dried and stored at −80 °C until analysis.

### 2.5. Chemical Analyses

#### 2.5.1. In Vitro Trial

Ground (1 mm) samples of diets and *Porphyra* sp. were analyzed for DM, organic matter (OM), and ether extract (EE) according to the AOAC [28]. After N determination using a Leco TruSpec CN^®^ (Leco Empowering Results, St. Joseph, MI, USA), crude protein (CP) was calculated by multiplying by 6.25. The neutral detergent fiber (NDF) and acid detergent fiber (ADF) were analyzed according to Van Soest et al. [29] using an ANKOM Model 220 Fiber Analyzer (Macedon, NY, USA) with α—amylase for NDF analysis in concentrate samples, and both NDF and ADF contents referred to ash-free weight. The acid detergent lignin (ADL) was determined by solubilization of cellulose with 72% sulphuric acid. The energy content was determined using an oxygen bomb calorimeter (PARR 1356, Biometer^®^). Free, protein-bound and fiber-bound condensed tannins in diet samples were sequentially extracted following the procedure described by Perez-Maldonado and Norton [30] using condensed tannins from quebracho powder (Roy Wilson Dickson Ltd., Mold, UK) as a standard.

The CH_4_ concentration was determined by gas chromatography (GC) using a HP Hewlett 5890, Packard Series II gas chromatograph (Waldbronn, Germany) equipped with a flame ionization detector (FID) and with an HP-INNOWAX crosslinked polyethylene glycol column (25 m × 0.2 mm × 0.2 μm). The carrier gas was He, and peaks were identified by comparison with a standard of known composition. A sample of 0.5 mL of gas was injected using a 1 mL Sample-Lock^®^ syringe (Hamilton, Nevada, USA). Total and individual VFA were analyzed by gas chromatography following the methodology described by Isac et al. [31]. The concentration of NH_3_–N was determined following the colorimetric method of Weatherburn et al. [32] using a spectrophotometer (Thermo Scientific, Genesys 10 μV Scanning, Madison, WI 53, 711 USA).

#### 2.5.2. In Vivo Trials

Samples of grass and clover silages were analyzed using NIRs by Eurofins Norway performed at Eurofins Agro Testing Wageningen (Binnenhaven 5, NL-6709 PD, Wageningen ISO/IEC 17025:2005 RvA L122) for DM, ash, CP and NDF content. Soybean meal pellets and crushed oats were analyzed by Eurofins Norway performed at Eurofins Food & Feed Testing Sweden (Lidköping) (Sjöhagsgatan 3, port 2, 531 40, Lidköping ISO/IEC 17025:2005 SWEDAC 1977) for DM, N by the Kjeldahl method, ash (2009/152/EU) and NDF (ISO/CD 16472). Ammonia and acetate concentrations in rumen samples were analyzed by following the respective methodologies described for in vitro trial.

### 2.6. Calculations and Statistical Analysis

The gas produced in batch cultures was adjusted to the exponential model: y = A (1 − e ^(−c (t-lag)^), where y represents the cumulative gas production (mL), t the incubation time (h), A the asymptote (total gas; mL) and c the organic matter degradation rate (h^−1^), and lag (h) is the initial delay in the onset of gas production. The parameters A, c and lag were estimated by an iterative least square procedure using the NLIN procedure of SAS (version 9.4; SAS Inst. Inc., Cary, NC, USA). The average gas production rate (AGPR; mL gas/h) is defined as the average gas production rate between the start of the incubation and T1/2 and was calculated as AGPR = A c/[2 (ln2 + c lag)]. Finally, the effective DM degradability (EDMD) was estimated assuming a rumen particulate outflow (Kp) of 0.03 per h according to the equation: EDMD = [(TDMD c)/(c + Kp)] e ^(−c lag)^.

The amount of VFA in the batch culture bottles after 24 h of incubation both for control and experimental diets was corrected for VFA in the rumen liquid used as inoculum. The amount of CH_4_ produced was calculated by multiplying the gas produced with its concentration in CH_4_.

The SPSS for Windows, version 19.0 (2010; SPSS Inc., Chicago, IL, USA) was used for in vitro trial data entry and statistical analysis. Data were analyzed using the ANOVA procedure considering diet as a fixed effect and incubation run as a random effect. When a significant (*p <* 0.05) effect of diet was found, post hoc comparison of means was made using the Tukey test. Differences were considered significant at *p <* 0.05 and trend at 0.05 < *p ≤* 0.10.

The Minitab for Windows version 17.3.0 (2016 Minitab Inc, Coventry, United Kingdom) was used for the in vivo trial data and statistical analysis. To determine in vivo CH_4_ production and lambs’ daily weight gain between different diets, data were analyzed using the ANOVA procedure considering block and diet as fixed effects and animals as random effects. When a significant effect of block or diet was found, post hoc comparison of means was made using the Tukey test. Differences were considered significant at *p <* 0.05 and trend at 0.05 < *p ≤* 0.10.

## 3. Results

### 3.1. In Vitro Trial

Diet only affected (*p =* 0.002) pH values (Table 3), being higher for the POR diet compared with the other studied diets. Diet tended to affect total VFA produced (*p =* 0.092) and isovalerate proportion (*p =* 0.082) but no effect of diet on other VFA molar proportions and the acetate to propionate ratio was observed.

No effect of diet was observed (Table 4) for ammonia concentration, CH_4_ produced (mL/g incubated DM) and the CH_4_ to VFA ratio.

The same pattern (no effect of diet) was observed for gas kinetic characteristics A and AGPR (Table 5), EDMD and TDMD, while diet tended to affect the fraction rate of gas production, c (*p =* 0.090).

### 3.2. In Vivo Trials

#### 3.2.1. In Vivo Experiment 1

The DM intake during the stay of sheep in respiration chambers (Table 6) was lower (*p =* 0.005) for animals receiving CON and SOY diets compared with CLO and POR diets. However, CH_4_ produced (L/kg DM intake) was not different depending on diet. There were no changes in animals’ live weight during the trial and thus no differences in live weight between sheep fed the four diets (results not shown).

#### 3.2.2. In Vivo Experiment 2

Lambs fed the CLO diet had higher (*p <* 0.001) DM, CP, NDF, digestible organic matter (DOM) and energy (FU) intake (Table 7) compared to lambs fed the CON diet. The lambs fed the SOY and POR diets had intermediate intakes, different from the animals receiving CON and CLO diets, except for FU, which was also higher for the POR diet. However, the growth rate was higher (*p <* 0.001) for SOY and POR diets compared to CON and CLO diets. The variation of growth rate was from 254 g per day for the SOY lambs to 161 g per day for the CLO lambs.

The average pH in sheep rumen was not affected by diet (Table 8). Concentration of ammonia was lower (*p <* 0.001) in the rumen of animals fed the CON diet compared to the animals fed the CLO, SOY and POR diets. The concentration of acetate in the rumen was affected by diet (*p =* 0.040), being higher for CON and POR diets compared with CLO and SOY diets.

## 4. Discussion

In the present work, we tested the hypothesis that *Porphyra* sp. can substitute soybean meal or clover silage as a protein source in sheep diets without affecting ruminal fermentation, growth rate and enteric CH_4_ production. *Porphyra* sp. was chosen as the seaweed source based on in vitro and in situ results by Tayyab et al. [7], Molina-Alcaide et al. [8] and Gaillard et al. [9]. Most commonly, *Porphyra* is known as nori and used as wrapping for sushi and eaten in soups in Japan. *Porphyra* is not cultivated or harvested on industrial scale for feed production in Europe and the use of *Porphyra* as a protein source for animal feed is therefore a challenge. However, the study of seaweed as a potential protein source in animal feed has global relevance, since the cultivation potential of seaweed is not in competition with current land use. Recently, research has focused on both brown and red seaweed species. While brown seaweeds, such as *Saccharina latissima*, contain phlorotannins, which prevent protein degradation, red species can be a source of bypass protein [7] and amino acids which can be utilized by ruminants [9]. Therefore, red seaweed species show promise as an alternative to more commonly used products such as soybean meal.

### 4.1. In Vitro

There were no differences among diets in total VFA production or in VFA profile. In contrast to De la Moneda et al. [19], the POR diet did not differ from the CON diet in VFA profile or the acetate/propionate ratio. Compared with the Porphyra diet used by De la Moneda et al. [19], the total VFA production in the POR diet was lower. However, the molar proportions of the individual VFA were similar to De la Moneda et al. [19], although the acetate/propionate ratio was slightly lower for POR (2.62 mol/mol) compared to [19] (3.2 mol/mol). The lack of differences in NH_3_ concentration between diets also indicates similar protein degradability. CH_4_ production did not differ between the diets and neither did the CH_4_/VFA ratio. The CH_4_/VFA ratio can be used as an indicator of the efficiency of ruminal fermentation, because CH_4_ is an energy loss for the host animal and VFA is used as an energy source and as substrates for the synthesis of other compounds [33].

In the present study, no differences in total gas production (A), c or AGPR were found. This is comparable to results by De la Moneda et al. [19], who found that diets including *Porphyra* sp. did not reduce the extent of fermentation compared to a control diet. This confirms that the in vitro degradation of the diets was similar for all four diets and that inclusion of white clover silage or *Porphyra* sp. can substitute soybean meal without compromising rumen fermentation.

### 4.2. In Vivo

The three protein sources studied were white clover silage, soybean meal and powdered *Porphyra* sp. The protein content differed between the sources, with soybean meal having higher proportions than *Porphyra* sp. and clover silage. Diets with protein enrichment (CLO, SOY and POR) were planned to be isoenergetic and isoproteinic, but chemical analysis showed that the SOY diet had a higher crude protein content compared to CLO and POR diets. As dietary protein concentration increases, organic matter, dry matter and hemicellulose digestibility increase due to enhanced microbial enzyme and VFA production [34]. Tayyab et al. [7] demonstrated, using in situ methods, that *Porphyra* sp. can supply the rumen with high amounts of degradable protein and supply a high amount of rumen escapable protein, digestible in the small intestine. The CP concentration in *Porphyra* sp. was compared with that of oil-seed by-products, such as sunflower meal and rapeseed meal. The total DM intake in the present trial showed a similar pattern in the two experiments. The lowest DM intake was observed for animals fed the CON diets, while the highest intake was observed for animals fed the CLO diet resulting in the highest CP intake. Both these diets were based on silages with crushed oats and vitamin–mineral mixture.

In an Australian experiment, enteric CH_4_ emissions were reduced by up to 67% when the red seaweed species *Asparagopsis armata* was included at 1.0% of the total diet offed to dairy cows [12]. Li et al. [21] investigated *A. taxiformis* in diets to sheep at inclusions of 0.5%, 1%, 2% and 3% and found up to an 80% reduction in enteric CH_4_ compared to a control diet. They suggest that the inhibition of methanogenesis was due to the presence of accumulated halogenated metabolites in the *Asparagopsis* biomass [21]. It is suggested that bromoform, one of four haloforms, the others being fluoroform, chloroform and iodoform, inhibits the methanogens in the final enzymatic step of methanogenesis in the rumen. Bromoform is also characterized as a volatile halogenated hydrocarbon and exhibits a wide range of pharmacological activities [35]. Brominated fatty acids are primarily synthesized by *Asparagopsis* [36]. Condensed tannins are suggested to reduce CH_4_ production, as demonstrated in a Canadian experiment with beef cattle [37]. In Table 2, the content of condensed tannins is reported, and it shows small differences between the diets.

In our experiment, the inclusion of seaweed constituted almost 10% of the total DM to ensure that the diets were both isoenergetic and isoproteinic. The high inclusion of *Porphyra* sp. was challenging due to palatability, requiring the preparation of a porridge to enable the animals to eat their daily ration. We did not find that *Porphyra* sp. influenced enteric CH_4_. De la Moneda et al. [19] concluded that none of the seven different seaweed species, including *Porphyra* sp., tested in vitro for enteric CH_4_ emission, had a noticeable anti-methanogenic effect. We measured values of enteric CH_4_ from sheep fed the diets in respiration chambers between 22.8–29.1 L/kg DM intake, which is in accordance with observations made in goats [16,38], sheep [39] and cattle [40]. The in vivo results supported the results of the in vitro study, which also showed no significant differences in CH_4_ emission between diets.

The protein value of *Porphyra* sp. was shown by Tayyab et al. [7] and Gaillard et al. [9] to be similar to, e.g., the protein value of rapeseed meal. The soybean pellets in this experiment had a higher protein concentration in DM than that of *Porphyra* sp. (54.5 and 37.1 g/kg fresh matter, respectively, Table 1) with a slight difference also in protein intake (223 and 202 g/d for SOY and POR, respectively) (Table 7). However, lambs fed these diets showed a similar growth rate (254 and 249 g/d, respectively). The high CP intake for animals fed the CLO diet was not reflected by a corresponding increase in daily growth rate (161 g/d) but was instead similar to the growth rate for animals fed the CON diet (163 g/d). The higher content of NDF in the CLO diet could explain the lack of response in growth rate. The digestibility of NDF varies among different forages and affects animal performance. Lactating dairy cows fed silages with similar NDF and CP contents but different NDF digestibility performed significantly differently in DM intake and milk yield [41]. It is suggested that inclusion of white clover in a diet increases voluntary feed intake due to a more rapid particle breakdown, faster rumen fermentation and lower mean rumen retention time [15]. For digestible organic matter intake and intake of energy (Feed Units), lambs fed the CON diet had lower intake than for the CLO, SOY and POR diets. The lower growth rate of lambs in the CLO diet does not seem to be explained by protein or energy intake. The higher growth rate of lambs in the SOY and POR diets is possibly due to a high degree of bypass protein entering the intestine from the protein sources soybean meal and *Porphyra* sp. The latter confirms the in vitro and in situ findings by Tayyab et al. [7] and Gaillard et al. [9].

White clover silage was not included in the studies of Tayyab et al. [7] and Gaillard et al. [9], and it is thus not possible to compare the present in vivo data with similar in vitro data. However, Damborg et al. [42] found similar in situ total tract protein degradability for white clover (911 g/kg) as Tayyab et al. [7] found for *Porphyra* sp. (906 g/kg). The white clover varieties in the Danish experiment were *Klondyke* and *Silvester*, while in the present study the variety *Litago* was used due to its adaption to arctic conditions. To our knowledge, the protein quality of *Litago* has not been studied, so it is not possible to confirm if it is of similar quality to the Danish varieties.

The nutritive value of proteins supplied in the diet depends on the proportions of amino acids that are digested and made available to the animal. The supply of essential amino acids relative to total energy supply influences the absolute and relative rate of protein and fat accretion in lambs. It is well documented that methionine and cysteine are the primary limiting amino acids for wool production and both wool production and live weight gain can be improved by supplementing sheep with methionine [43]. Gaillard et al. [9] showed that most of the seaweeds investigated (*Porphyra* incl.) were deficient in essential amino acids compared to soybean, except for sulphur-amino acids. Most amino acids are degraded in the rumen and, to characterize a feedstuff, amino acids that escape to the small intestine are important [44]. Gaillard et al. [9] concluded that *Porphyra* can be considered as relevant source of protein for ruminants based on the amount of amino acids and their degradability but should be harvested during spring when higher quantities of amino acids are present.

The diet effects of ammonia and acetate concentrations in the rumen were different for the in vivo and in vitro experiments. While there was no response to diet in vitro, the CON diet resulted in lower ammonia concentration compared to the three protein-supplemented diets in vivo. The ammonia concentration in the CON diet was similar to all the observed in vitro concentrations (around 10 mg/100 mL). This could be explained by the lower protein content in the CON diet compared to the other three diets, indicating that in vitro methods do not necessarily mirror in vivo ammonia. A review by Eschenlauer [45] concludes that different methodologies and substrate concentrations explain different rates of ammonia production in the rumen of sheep.

## 5. Conclusions

Both in vivo and in vitro results indicate that *Porphyra* sp. does not act as a tool to reduce enteric methane emissions. We show that *Porphyra* sp. has a protein value similar to high quality protein sources, such as soybean meal. This in vivo study confirms that *Porphyra* sp. is a comparably effective protein source when compared with soybean meal. On the other hand, *Porphyra* sp. is not effective in reducing enteric methane. The use of seaweed species as a protein source in animal production faces some practical and economic challenges before it can substitute soybean meal and be implemented in the daily diet of ruminants.

## Figures and Tables

**Table 1:** Average chemical composition (g/kg DM) of diet ingredients.

Item.	Grass Silage	White Clover Silage	Crushed Oats	Soybean Meal Pellets	*Porphyra* sp.
DM, g/kg fresh	383	352	943	932	947
Ash	60.9	86.8	28.0	60.0	21.6
Crude protein	137	137	235	545	371
NDF	521	433	115	120	58
DOM ^1^	707	721	972	929	898
FU ^2^/kg DM	0.83	0.83	1.09	1.10	0.80

^1^ DOM: in vitro Digestible organic matter; ^2^ FU Scandinavian Feed Unit: 1 FU = 7.89 MJ net energy.

**Table 2:** Ingredients (g/kg fresh matter) and chemical composition (g/kg DM) of control and experimental diets.

Diet ^1^	CON	CLO	SOY	POR
Ingredients				
Grass silage	922	352	835	833
Crushed oats	62	46	55	56
Clover silage		590		
Soybean meal			96	
*Porphyra* sp.				97
Mineral–vitamin mixture	16	12	14	14
Chemical composition of dry matter				
Organic matter	918	927	933	927
Crude protein	111	125	137	126
Ether extract	37.2	36.8	36.5	34.5
Neutral detergent fiber	511	516	491	502
Acid detergent fiber	274	274	248	270
Acid detergent lignin	25.6	33.8	23.2	22.8
Gross energy, MJ/kg DM	17.2	16.7	17.3	17.0
Protein bound condensed tannins	0.158	0.281	0.239	0.224
Free condensed tannins	0.307	0.294	0.296	0.240
Fibre bound condensed tannins	9.44	8.43	8.56	10.26
Total condensed tannins	9.91	9.01	9.10	10.7

^1^ CON: control diet; CLO: diet including clover silage; SOY: diet including soybean meal; POR: diet including *Porphyra* sp.

**Table 3:** Average values of pH, total volatile fatty acids (VFA) produced and molar proportions of individual VFA and acetate:propionate ratios after 24 h of incubation of the experimental diets in batch cultures inoculated with ruminal microorganisms from sheep.

Diet ^1^	pH	VFA (mmol)	Molar Proportions (mol/100 mol)						Acetate/Propionate (mol/mol)
			Acetate	Propionate	Butyrate	Isobutyrate	Isovalerate	Valerate	
CON	6.76 ^a^	2.29	62.5	24.1	10.2	0.263	1.51	1.43	2.67
CLO	6.76 ^a^	2.46	61.9	24.2	10.5	0.289	1.59	1.49	2.65
SOY	6.75 ^a^	2.43	62.2	24.1	10.1	0.288	1.73	1.51	2.66
POR	6.82 ^b^	2.35	62.1	24.4	10.2	0.256	1.62	1.45	2.62
SEM ^2^	0.012	0.023	0.944	0.952	0.365	0.023	0.083	0.140	0.150
*p* value	0.002	0.092	0.344	0.857	0.203	0.580	0.082	0.163	0.928

^1^ CON: control diet; CLO: diet including clover silage; SOY: diet including soybean meal; POR: diet including *Porphyra* sp.; ^2^ SEM: standard error of the mean; ^a,b^ Means that do not share a letter are significantly different.

**Table 4:** Average values of NH_3_–N concentrations (mg/100 mL), production of methane (mL/g DM), and methane/total volatile fatty acids (methane/VFA mol/mol) ratios after 24 h of incubation of the experimental diets in batch cultures inoculated with ruminal microorganisms from sheep.

Diet ^1^	NH_3_	Methane	Methane/VFA
CON	10.5	69.6	12.3
CLO	9.95	63.8	10.6
SOY	11.8	72.7	12.2
POR	11.0	72.5	12.5
SEM ^2^	0.760	4.26	0.854
*p* value	0.345	0.253	0.955

^1^ CON: control diet; CLO: diet including clover silage; SOY: diet including soybean meal; POR: diet including *Porphyra* sp.; ^2^ SEM: standard error of the mean.

**Table 5:** Gas production kinetics (A, c and AGPR), effective dry matter degradability (EDMD) and true dry matter digestibility (TDMD) after 72 h of incubation of the experimental diets in batch cultures inoculated with ruminal microorganisms from sheep.

Diet ^1^	Parameters of Gas Production Kinetics ^2^				
	A (mL)	c (h^−1^)	AGPR (mL/h)	EDMD (g/kg) ^3^	TDMD (g/kg) ^4^
CON	107	0.058	4.50	57.8	87.7
CLO	107	0.097	4.80	58.9	87.3
SOY	110	0.058	4.57	58.1	88.3
POR	107	0.061	4.67	58.6	87.6
SEM ^5^	0.617	0.002	0.015	0.440	0.183
*p* value	0.230	0.090	0.102	0.814	0.319

^1^ CON: control diet; CLO: diet including clover silage; SOY: diet including soybean meal; POR: diet including *Porphyra* sp.; ^2^ A: asymptotic gas production; c: fractional rate of gas production; AGPR: average gas production rate. The values of lag were 0 for all samples. ^3^ EDMD: Effective dry matter degradability for a rumen particulate outflow of 0.03/h; ^4^ Calculated as described by Van Soest et al. [24]; ^5^ SEM: standard error of the mean.

**Table 6:** Average daily DM intake (g/day) and enteric methane production (L CH_4_/kg DM) in sheep fed the experimental diets in in vivo Experiment 1.

Diet ^1^	DM	CH_4_
CON	1343 ^a^	22.8
CLO	1544 ^b^	29.1
SOY	1466 ^ab^	24.6
POR	1507 ^b^	24.6
SEM ^2^	21.3	1.26
*p* value	0.005	0.327

^1^ CON: control diet; CLO: diet including clover silage; SOY: diet including soybean meal; POR: diet including *Porphyra* sp.; ^2^ SEM: standard error of the mean; ^a,b^ Means that do not share a letter are significantly different.

**Table 7:** Average DM, CP, NDF, DOM (g/d) and feed unit (FU) intake and growth rate (g/d) of lambs fed the experimental diets in in vivo Experiment 2.

Diet ^1^	DM	CP	NDF	DOM	Feed Units ^3^	Growth Rate
CON	1395 ^a^	115 ^a^	712 ^a^	1046 ^a^	1.17 ^a^	163.3 ^a^
CLO	1967 ^c^	246 ^d^	1015 ^c^	1222 ^b^	1.38 ^bc^	160.5 ^a^
SOY	1625 ^b^	223 ^c^	798 ^b^	1251 ^b^	1.42 ^c^	253.8 ^b^
POR	1600 ^b^	202 ^b^	803 ^b^	1227 ^b^	1.34 ^b^	249.0 ^b^
SEM ^2^	11.6	1.69	6.13	127.8	0.14	12.3
*p* value	<0.001	<0.001	<0.001	<0.001	<0.001	<0.001

^1^ CON: control diet; CLO: diet including clover silage; SOY: diet including soybean meal; POR: diet including *Porphyra* sp.; ^2^ SEM: standard error of the mean; ^3^ FU Scandinavian Feed Unit—1 FU = 7.89 MJ net energy; ^a,b,c^ Means that do not share a letter are significantly different.

**Table 8:** Average values of pH, concentration of ammonia (mg/100 mL) and of acetate (mmol/L) in the rumen of sheep fed the experimental diets in in vivo Experiment 2.

Diet ^1^	pH	NH_3_	Acetate
CON	7.21	10.2 ^a^	44.5 ^b^
CLO	7.60	23.0 ^b^	38.0 ^a^
SOY	7.50	26.1 ^b^	38.4 ^a^
POR	7.09	21.4 ^b^	47.5 ^b^
SEM ^2^	0.111	1.06	5.12
*p* value	0.357	0.001	0.040

^1^ CON: control diet; CLO: diet including clover silage; SOY: diet including soybean meal; POR: diet including *Porphyra* sp.; ^2^ SEM: standard error of the mean; ^a,b^ Means that do not share a letter are significantly different.
